# Drought, Salinity, and Low Nitrogen Differentially Affect the Growth and Nitrogen Metabolism of *Sophora japonica* (L.) in a Semi-Hydroponic Phenotyping Platform

**DOI:** 10.3389/fpls.2021.715456

**Published:** 2021-10-01

**Authors:** Jing Tian, Yue Pang, Zhong Zhao

**Affiliations:** ^1^ College of Forestry, Northwest A&F University, Yangling, China; ^2^ Research Center for the Conservation and Breeding Engineering of Ancient Trees, Yangling, China; ^3^ Key Comprehensive Laboratory of Forestry, Yangling, China

**Keywords:** *Sophora japonica*, drought, salinity, low nitrogen, *AMTs* and *NRTs*, nitrogen uptake and metabolism

## Abstract

Abiotic stresses, such as salinity, drought, and nutrient deficiency adversely affect nitrogen (N) uptake and assimilation in plants. However, the regulation of N metabolism and N pathway genes in *Sophora japonica* under abiotic stresses is unclear. *Sophora japonica* seedlings were subjected to drought (5% polyethylene glycol 6,000), salinity (75mM NaCl), or low N (0.01mM NH_4_NO_3_) for 3weeks in a semi-hydroponic phenotyping platform. Salinity and low N negatively affected plant growth, while drought promoted root growth and inhibited aboveground growth. The NH_4_^+^/NO_3_^−^ ratio increased under all three treatments with the exception of a reduction in leaves under salinity. Drought significantly increased leaf NO_2_^−^ concentrations. Nitrate reductase (NR) activity was unaltered or increased under stresses with the exception of a reduction in leaves under salinity. Drought enhanced ammonium assimilation with increased glutamate synthase (GOGAT) activity, although glutamine synthetase (GS) activity remained unchanged, whereas salinity and low N inhibited ammonium assimilation with decreased GS activity under salt stress and decreased GOGAT activity under low N treatment. Glutamate dehydrogenase (GDH) activity also changed dramatically under different stresses. Additionally, expression changes of genes involved in N reduction and assimilation were generally consistent with related enzyme activities. In roots, ammonium transporters, especially *SjAMT1.1* and *SjAMT2.1a*, showed higher transcription under all three stresses; however, most nitrate transporters *(NRTs)* were upregulated under salinity but unchanged under drought. *SjNRT2.4*, *SjNRT2.5*, and *SjNRT3.1* were highly induced by low N. These results indicate that N uptake and metabolism processes respond differently to drought, salinity, and low N conditions in *S. japonica* seedlings, possibly playing key roles in plant resistance to environmental stress.

## Introduction

Excessive drought, salinity, and nutrient limitations are the main forms of abiotic stresses that significantly affect plant growth and development. Currently, it is estimated that more than 6% of the world’s land and 20% of the total irrigated area are affected by salt with major affliction in the Asian countries (Munns and Tester, 2008; FAO and ITPS, 2015). Forest trees are expected to face more frequent and severe droughts as a result of climate change (Plattner and GianKasper, 2014). Such soil conditions will further reduce nitrogen (N) availability and accelerate N limitation during the vegetative growth periods (Plattner and GianKasper, 2014). In agriculture, N limitation is overcome by the application of N fertilizers. However, trees usually have a high distribution on marginal soils with low N availability and are rarely managed. Plants, particularly long-living trees, have evolved various strategies to cope with different stressful conditions. N is a constituent of many plant cell components, such as chlorophyll, nucleic acids, proteins, amino acids, and some hormones which are involved in the ability of plants to resist abiotic stresses through different mechanisms (Kamel, 2012; Rais et al., 2013). Exploring the N acquisition strategy and improving the N uptake efficiency under stressful conditions are of great significance for maintaining the healthy growth of trees, breeding resistant varieties, and providing feasible management methods.

Nitrogen is one of the essential macronutrients for plant growth and development. Higher plants absorb inorganic NH_4_^+^ and NO_3_^−^ *via* specific ammonium *(AMTs)* and nitrate transporters (*NRTs*; Kaiser et al., 2002; Wang et al., 2018b). Subsequently, nitrate assimilation depends on two enzymes, nitrate reductase (NR) and nitrite reductase (NiR), to convert NO_3_^−^ to NH_4_^+^ (Xu et al., 2012). More than 95% of ammonium assimilation is performed *via* glutamine synthetase (GS) and glutamate synthase (GOGAT) in plants (Suárez et al., 2002). NH_4_^+^ can also be assimilated by the conversion of a-oxoglutarate to glutamate *via* glutamate dehydrogenase (GDH). N transport and assimilation processes are regulated by internal and external signals, leading to differences in N metabolism of plants in response to different environmental stresses. The variation in nitrogen concentrations, enzyme activities, and transcript levels of genes related to N utilization can reflect the accumulation strategies of nitrogen under different stresses.

Drought and excessive amounts of salt are known to negatively affect plant growth and productivity (Boyer, 1982; Park Williams et al., 2012; McDowell et al., 2018). Previous studies have demonstrated that *Sophora japonica* showed growth limitation, as indicated by declines in the stem base diameter, height increment, chlorophyll pigment contents, osmotic regulator contents, and altered antioxidative enzyme activities under both drought and salinity (Quy et al., 2017; Lu et al., 2018). Reductions in N uptake and metabolism have been observed in many tree and crop species (GeBler et al., 2005; Zhang et al., 2014; Ahanger et al., 2017; Ashraf et al., 2018). For instance, salinity decreased the net N uptake rate and total N content in *Populus canescens* (Dluzniewska et al., 2007). Drought stress inhibited ammonium and nitrate uptake in *Malus prunifolia* and beech (Rennenberg et al., 2006; Huang et al., 2018) and decreased NR, GS, and GOGAT activities in *M. prunifolia* (Huang et al., 2018). Genes involved in N uptake and metabolic processes also play key roles in plant resistance to extreme conditions. Meng et al. (2015) reported that *AMT1.2* may play key roles in NH_4_^+^ uptake under both drought and salt stress in *Populus*. The enzyme-encoding genes *GS*, *GOGAT*, and *GDH* were induced by drought, cold, or heat stress in tomato (Liu et al., 2016). Co-overexpression of *GS1.1* and *GS2* enhanced tolerance to osmotic and salinity stress in rice (James et al., 2018). The physiological and molecular responses of N uptake and metabolism process to abiotic stress are widely studied in annual plants such as crops or model genera such as *Arabidopsis* and *Populus*, whereas information on trees is limited. It is worth noting that although some effects are shared between salinity and drought stress, as both stresses lower the soil water potential, differences in the potential regulatory mechanisms exist, which eventually affect N absorption, reduction, and assimilation differently. Generally, drought reduces N uptake and translocation, as well as NR and GS activities, because of restricted photosynthetic and transpiration rates (Bowen et al., 2018; Iqbal et al., 2020). Apart from the results caused by limited water absorption, salt stress adds additional negative effects. For example, high concentrations of Na^+^ and Cl^−^ cause ion toxicities and ionic imbalances, which can directly inhibit N transport and assimilation processes (Flores et al., 2000; Khare et al., 2020). Our previous research showed that salinization, 50mM NaCl application for 10days, prominently affected the root system of *S. japonica* seedlings, causing a less developed root system, but under drought, the roots thrived (Quy et al., 2017). Therefore, it is necessary to verify the underlying regulatory differences between drought and salinity, particularly, in the N utilization process.

*Sophora japonica,* also known as Chinese scholar tree, is a leguminous tree species native to China and is cultivated in Vietnam, Japan, Korea, and other regions. High stress tolerance contributes to its long lifespan of thousands of years. *Sophora japonica* has been widely planted recently because of its medicinal and economic value. Most legumes can alleviate N limitation by fixing atmospheric N *via* nodules in soils, but *S. japonica* is an exception that lacks this ability, so its growth is inhibited under low N conditions unlike that of other legume species such as *Robinia pseudoacacia* that can fix N (Yin et al., 2006; Wang et al., 2018a). However, little is known about the mechanisms of N uptake and metabolism of *S. japonica* under low N conditions. Few have studied the molecular regulation of N utilization due to the lack of genomic resources for *S. japonica* in the past, but we have discovered related candidate genes through transcriptome sequencing (Tian et al., 2021). This study provides the basis to study the N uptake and assimilation process of *S. japonica* in response to low N conditions.

In this study, to understand the morphological, physiological, and molecular responses of *S. japonica* to drought, salinity, and low N stresses, we performed a controlled experiment using a semi-hydroponic phenotyping platform that is quite efficient for studying root architecture (Chen et al., 2011). Morphological (shoot and root traits), physiological (photosynthesis, chlorophyll content, total N concentration, accumulation of NH_4_^+^, NO_3_^−^, and NO_2_^−^, and activities of N metabolism enzymes, including NR, NiR, GS, GOGAT, and GDH), and molecular (transcript levels of genes involved in N uptake and metabolism) indices were assessed. It was hypothesized that (1) the process of N uptake and metabolism would change significantly in response to the three stress treatments and (2) *Sophora japonica* would exhibit differential root growth under drought and salinity stresses. Studying the effects of different stresses on N uptake and metabolism is of particular importance for the generation of resistant varieties of *S. japonica* using transgenic approaches to improve N use efficiency and tolerance to abiotic stresses.

## Materials and Methods

### Plant Materials and Treatments

**Figure 1:**
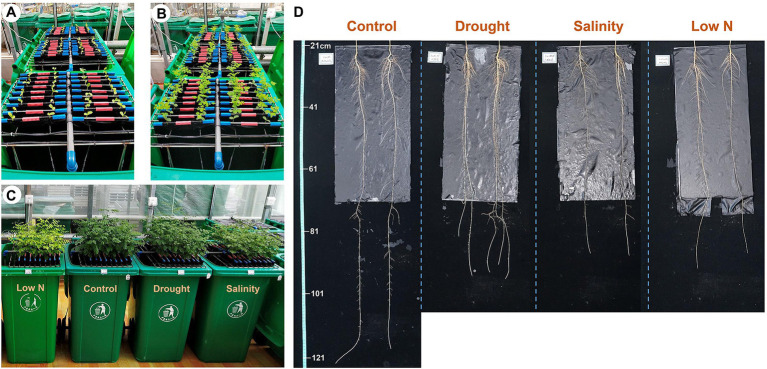
*Sophora japonica* seedlings grown in a semi-hydroponic phenotyping platform, 1day **(A)** and 7days **(B)** after transplanting; aboveground growth **(C)** and root morphology **(D)** of plants under control, drought, salinity, and low N treatment at harvest.

Seeds of *S. japonica* were purchased from Shaanxi Sanyuan Gold Seed Industry Technology Co., Ltd., Yangling Branch. After the seeds germinated, uniform seedlings about 3days old were transferred to a semi-hydroponic phenotyping platform, which was established as Chen et al. (2011) described (Figures 1A,B). Four bins, each accommodating 36 plants, were placed in a greenhouse (natural light, day/night 28/20°C, and 80% relative humidity) at Northwest A&F University, Yangling (34°16’N, 108°4''E). Each bin contained 30L of nutrition solution (Meng et al., 2015) supplemented with 1mM NH_4_NO_3_ as the N source. The solution was renewed weekly throughout the experiment.

We set up three stress treatments (one treatment one bin) in this study. The saplings that grew normally for 7weeks were used as controls. The drought, salinity, and low N treatments commenced 4weeks after transfer and lasted for 3weeks before harvesting. Mild drought stress was applied by adding 5% polyethylene glycol (PEG) 6,000 to the solution. Moderate salt stress was increased gradually by applying 25, 50, and finally 75mM NaCl on the first, fourth, and seventh days of treatment to avoid early lethal damage. Then, NaCl concentration was maintained at 75mM during the remaining 2weeks until harvest. Low N stress was initiated by setting the N concentration at 0.01mM. As *Populus* is the model tree species used in studies on woody plant molecular biology, the choice of PEG-6000, NaCl, and NH_4_NO_3_ concentrations in this study was based on our previous studies on *Populus simonii* stress resistance in a hydroponic system (Meng et al., 2015, 2016). Samples were harvested when seedlings showed obvious phenotypic differences among treatments at the end of third week after the stresses began.

At harvest, seedlings that were infected with pests or disease or had mechanical damage caused by experimental operations during the stress period were excluded from each treatment. As a result, approximately 80% of the plants were collected for further measurements. Shoots and roots were harvested separately. The morphological indices were determined with eight replicates (one plant one replicate) per treatment. Three biological replicates, each containing a pool of four different plants, were rapidly frozen in liquid nitrogen for all physiological and molecular assays.

### Net Photosynthetic Rate and Chlorophyll Content

The leaf net photosynthetic rate was measured 1day before harvest from 9:00 to 11:00h on the fully expanded leaves of six plants per treatment using a Li-6400 portable photosynthesis system (Li-Cor, Lincoln, NE, United States). The measurement conditions were as follows: attached LED light source 1,000μmol photon m^−2^s^−1^, CO_2_ concentration in the chambers 400μmolmol^−1^, and air flow 500μmols^−1^. The chlorophyll content of 12 plants in each treatment was measured with a SPAD 502 plus chlorophyll meter (Minolta SPAD 502 Meter).

### Shoot Traits, Root Morphology, and Total N Concentrations

The shoot height and stem base diameter were measured using a ruler. The compound leaf number (CLN) was recorded simultaneously. All the lobules of the 4–7th compound leaves from the bottom were flattened on mesh paper (1cm×1cm), photographed with a camera, and processed in Photoshop CS (Adobe Systems, San Jose, United States) to obtain the leaf area (LA). These leaves were also collected to calculate the leaf water content (LWC) as follows:

LWC = (FW−DW)/DW

where FW is the fresh weight of leaves obtained immediately after harvest and DW is the dry weight of leaves obtained after drying at 70°C to constant weight. Root systems were photographed using a portable photo box first. The primary root length was measured manually. Then, subsamples of roots were cut into three sections (0–20, 20–40, and >40cm) according to the root depth along the glass sheet. All root section samples were scanned and analyzed by a WinRHIZO root analyzer system (WinRHIZO version 2007b, Regent Instruments Canada, Montreal, Canada) in the laboratory. Root morphology parameters, such as root length, root surface area (TRSA), and root diameter were generated. After the above determinations, subsamples of roots from one plant were combined into one root sample. Aerial biomass (AB) and total root dry mass were recorded after oven-drying at 70°C for 72h. Leaves and roots were then ground into powder for measurement of the total N concentration by dry combustion using an elemental analyzer (Vario MAX CN Elemental Analyzer, Elementar, Germany).

### Determination of NH_4_^+^, NO_3_^−^, and NO_2_^−^ Concentrations

Concentrations of NH_4_^+^ in the leaves and roots were calculated based on the Berthelot reaction (Bräutigam et al., 2007). The concentration of NO_3_^−^ was analyzed as described by Patterson et al. (2010). The concentration of NO_2_^−^ was quantified as described by Ogawa et al. (1999). The detailed laboratory experimental processes were reported in our previous study (Meng et al., 2015).

### Determination of Enzyme Activity Involved in N Assimilation

The activities of NR and GOGAT in the roots and leaves were assayed according to the methods previously described (Högberg et al., 1986; Singh and Srivastava, 1986), and they were defined as the catalytic reduction in the amount of nicotinamide adenine dinucleotide (NADH) per gram fresh weight sample per minute. The activity of NiR was defined as the reduction in the amount of NO_2_^−^ per gram fresh weight sample per hour (Seith et al., 1994). GS activity was analyzed spectrophotometrically (Yu and Zhang, 2012) and defined as the amount of γ-glutamyl isohydroxamic acid produced per gram fresh weight sample per minute. GDH activity was measured using a physiological assay kit (GDH-1-Y, Suzhou Keming Biological Technology Co., Ltd., Suzhou, China) according to the manufacturers’ recommendations.

### Identification of Genes Involved in N Uptake and Assimilation

In our previous study, we conducted RNA-seq of *S. japonica* roots exposed to 20% PEG-6000 induced drought stress under normal N and N starvation conditions for 3h (Tian et al., 2021). Based on the transcriptomic annotation information, unigenes involved in N transport and metabolism were obtained. These protein sequences were then submitted to the *Arabidopsis* genome database^1^ for BLAST homology. When multiple sequences were annotated to the same homologous gene of *Arabidopsis thaliana*, only the longest was retained. Unigenes with an expression level lower than five fragments per kilobase per million mapped reads (FPKM) and no differential expression were also excluded. A phylogenetic tree of 22 identified NRT protein sequences from *S. japonica* and 61 NRT family members from *Arabidopsis* was constructed *via* the neighbor-joining (1,000 bootstrap replicates) method with MEGA 7.0 software (Center for Evolutionary Medicine and Informatics, Tempe, AZ, United States). According to the clustering results (Supplementary Figure S1), 12 *NRTs* were clustered together with those in *Arabidopsis* that have been proven to transport NO_3_^−^. Then 12 *NRTs*, as well as four *AMTs* and nine genes encoding N assimilation including one *NR*, one *NiR*, three *GSs*, two *GOGATs*, and two *GDHs*, were selected for quantitative real-time PCR (qRT-PCR) analysis in this experiment (Table 1). We provisionally named these genes based on the identities in protein BLAST results between *S. japonica* and *A. thaliana* sequences. The protein sequences of *S. japonica* are provided in Supplementary Table S1, and related gene accession numbers in *Arabidopsis* are listed in Supplementary Table S2.

**Table 1:** The information of genes related to nitrogen uptake and metabolism in *Sophora japonica*.

Unigene ID	Name	Homologous gene of *Arabidopsis thaliana*	Forward primer (5'-3')	Reverse primer (5'-3')
c1228676.graph_c1	*SjNPF7.3*	*NPF7.3 (NRT1.5)* [AT1G32450.1]	CAAATCCCTCCTGCCAGCATGAC	GCCAATTCTTCGTAGCTCGCTAGG
c1230955.graph_c0	*SjNPF2.11*	*NPF2.11(NRT1.10)* [AT5G62680.1]	CCTCGGGTAACCAATTCCCTGTTG	CCCTGAGAACATGAGAAGCCTTGC
c1241780.graph_c0	*SjNPF2.13*	*NPF2.13 (NRT1.7)* [AT1G69870.1]	GCTTCTGCCTCTGCCTCTTCTTG	CCATCCTCCAGGCTGCTTCTTG
c1250133.graph_c0	*SjNPF4.4*	*NPF4.4 (NRT1.13)* [AT1G33440.1]	TTCCCAGAGCCACCAAGTAGAGAG	TCAGTTCAAGCCCATCTTCCACAG
c1253196.graph_c0	*SjNPF4.3*	*NPF4.3 (NRT1.14)* [AT1G59740.1]	AGCCACTCCCTAATGCCACCAA	TCCTCAGTTGAAGCCACCCTCA
c1254472.graph_c0	*SjNPF4.6*	*NPF4.6 (NRT1.2)* [AT1G69850.1]	CATGGCCGTGGCTGCTCTTG	AGGTAGTGGCTTGGTTGCATCATC
c1255452.graph_c1	*SjNPF2.9*	*NPF2.9 (NRT1.9)* [AT1G18880.1]	CGACAGGCGTATTGGGCATAGC	CGGGTGAGCCTTCGGAGAAAAG
c1261082.graph_c0	*SjNPF6.3*	*NPF6.3 (NRT1.1)* [AT1G12110.1]	CAGTATCACAGGCGACCACCATG	CGAAGAAGACGGTCAGCGATGC
c1263149.graph_c0	*SjNPF1.2*	*NPF1.2 (NRT1.11)* [AT1G52190.1]	AACCTAACCCAACAGCGACGAATC	TAGCCACAGCAACCCAGATACTCC
c1213028.graph_c1	*SjNRT2.4*	*NRT2.4* [AT5G60770.1]	TTCTCCACATCCGCAGGTCTCTC	AGGTCATTTGAGGGAGGGAGGAAC
c1215796.graph_c0	*SjNRT2.5*	*NRT2.5* [AT1G12940.1]	AAGCCAGTGAAGAAGCGTACAAGG	TTGCCTCTGCCTCTCTCATCCTC
c1247312.graph_c0	*SjNRT3.1*	*NRT3.1* [AT5G50200.1]	GACCTGCCAGCATAAGATCGTAGC	CGTCGTTGGAGTCATGAGCGTAG
c1238615.graph_c0	*SjAMT1.1*	*AMT1.1* [AT4G13510.1]	TCACGGCGTTGTTTGCTAAGGAG	GCTTCCCACCACCACCCATAAAC
c1241960.graph_c0	*SjAMT1.2*	*AMT1.2* [AT1G64780.1]	CCATAGCCGCAGCAGGAATCAC	GCGAGACAATGGGATAGACGAAGC
c1244303.graph_c0	*SjAMT2.1a*	*AMT2.1* [AT2G38290.1]	GCAGCCCATCCTTGAACCAGAC	AGCCTCCTCGTGTGGACTTGG
c1245778.graph_c1	*SjAMT2.1b*	*AMT2.1* [AT2G38290.1]	GCCTCCTCACAGGTCTCTTAGC	GCACACCACCACTTCCACCATA
c1249066.graph_c1	*NR*	*NR* [AT1G77760.1]	CCAATAGGCAGCGAGTCCAAGTG	CGAGCAGCGTGGATGAAGTGG
c1243496.graph_c0	*NiR*	*NiR* [AT2G15620.1]	GTTCGCTTTCTCACCCCTCCATG	TTCCTCCACTCTCGGCTCCAAC
c1249069.graph_c1	*GS1.2*	*GS* [AT1G66200.1]	GTTGGTCCTTCGGTTGGCATCTC	AGCACCAGCACCATTCCAATCAC
c1259891.graph_c0	*GS1.1*	*GS* [AT5G37600.1]	ACTACAGCACCAAGTCCATGAG	GCCTTCTCCATAAGCAGCAATG
c1260647.graph_c1	*GS2*	*GS* [AT5G35630.1]	CTTCGCCACCAGGTCCACATTAG	GCAACCACGGTTAGCCACTCC
c1245882.graph_c1	*Fd-GOGAT*	*Fd-GOGAT* [AT5G04140.1]	AAGAGGAGCCTGCGGTGTTG	CACGATGTTCCATACAGCCAAGAG
c1264570.graph_c0	*NADH-GOGAT*	*NADH-GOGAT* [AT5G53460.1]	GTGATGCCAGGACAGAGGAATGC	CCGTGCTGTGGGAACTATGCTTAG
c1257681.graph_c0	*GDH2*	*GDH2* [AT5G07440.1]	CCTAGTCCTGTGGCAGCCTCTC	ACGGGCATTCACCAGCAGTTG
c1262840.graph_c1	*GDH1*	*GDH1* [AT5G18170.1]	ACGAGAACATCGCAGTCTTCAACC	CCCAAGCCTACTCAAGCATTCCAG
--	*18s rRNA*	*--*	ATAAACGATGCCGACCAG	GATGGCTGGAACAGAACTT

NPF, nitrate transporter 1(NRT1); NRT2, nitrate transporter 2; NRT3, nitrate transporter 3; AMT1, ammonium transporter 1; AMT2, ammonium transporter 2; NR, nitrate reductase; NiR, nitrite reductase; GS, glutamine synthetase; Fd-GOGAT, ferredoxin-dependent glutamate synthase; NADH-GOGAT, NADH-dependent glutamate synthase; and GDH, glutamate dehydrogenase.

### Analysis of Transcript Levels for Genes Involved in N Uptake and Assimilation

Total RNA was extracted and purified from leaves and roots with a plant RNA extraction kit (R6827, Omega Bio-Tek, GA, United States). Concentrations of RNA samples were estimated using NanoDrop 20000 (Thermo Scientific, Pittsburgh, PA, United States). Only when OD260/280 ratios were between 1.8 and 2.2 and OD260/230 ratios were higher than 2.0, could the samples be used for further experiments. First-strand cDNA synthesis was performed using EasyScript® One-Step gDNA Removal and cDNA Synthesis SuperMix (TransGen Biotech, Beijing, China) according to the manufacturer’s instructions using an anchored oligo (dT)_18_ primer and 500ng of total RNA. Reactions were performed on a LightCycler® 96 real-time PCR system (Roche, Mannheim, Germany) using 2×SYBR real-time PCR premixture (Bioteke, Beijing, China) in a 20μl reaction under the following conditions: one cycle of 150s at 95°C, followed by 40cycles at 95°C for 10s, 58°C for 20s, and 72°C for 30s. The primers are listed in Table 1. *18s rRNA* was used as the reference gene. For each of the selected genes, three biological replicates with three technical replicates were assayed. The relative expression levels were calculated using the 2^−ΔΔCT^ method (Livak and Schmittgen, 2001).

### Statistical Analysis

Statistical tests were performed with SPSS 23 (SPSS Inc., Chicago, IL, United States) by one-way ANOVA. The normality of all data was tested prior to further analysis. Differences between means were determined by Tukey’s test at the *p*<0.05 probability level.

## Results

### Plant Growth Parameters

After 3weeks of exposure to drought, salinity, and low N stresses, the shoot traits of *S. japonica* seedlings were indicative of growth inhibition under all three stresses, mainly showing decreases in the shoot height (SH) and leaf water content (LWC; Figure 1C; Table 2). The stem base diameter (SBD) was only reduced under salinity stress, while the CLN was only reduced under the low N treatment. LA and AB were negatively affected by both salinity and low N but had no change under drought (Table 2).

**Table 2:** Effects of drought, salinity, and low nitrogen on growth parameters of *S. japonica*.

Traits	Control	Drought	Salinity	Low N
Shoots traits				
SH (cm)	22.6±0.37 a	19.8±0.66 b	16.7±0.32 c	16.5±0.43 c
SBD (mm)	2.55±0.09 a	2.45±0.08 a	2.12±0.07 b	2.27±0.07 ab
LA (cm^2^)	85.8±2.83 a	81.1±3.12 a	67.45±2.36 b	53.00±2.99 c
CLN	10.0±0.27 ab	10.4±0.26 a	9.0±0.33 bc	8.6±0.26 c
LWC (mLg^−1^DW)	3.28±0.11 a	2.74±0.15 b	2.73±0.09 b	2.56±0.29 b
AB (g DW)	0.86±0.03 a	0.82±0.01 a	0.61±0.02 b	0.54±0.01 b
Roots traits				
PRL (cm)	92.4±2.26 a	71.1±2.75 b	64.0±1.56 b	69.3±2.49 b
ARD (mm)	0.48±0.008 a	0.46±0.021 a	0.48±0.029 a	0.49±0.019 a
TRL (m)	8.29±0.54 b	10.5±0.72 a	7.27±0.41 b	5.02±0.38 c
TRSA (cm^2^)	109.2±6.63 b	144.5±5.82 a	96.6±5.44 bc	75.2±5.67 c
TRV (cm^3^)	1.36±0.16 ab	1.66±0.13 a	1.11±0.09 b	0.91±0.08 b
TRDW (g DW)	0.54±0.03 b	0.69±0.03 a	0.40±0.02 c	0.41±0.01 c
RSM	0.63±0.03 b	0.84±0.03 a	0.65±0.03 b	0.76±0.02 a
RL-upper (m)	4.84±0.24 b	7.25±0.35 a	4.98±0.27 b	3.25±0.25 c
RA- upper (cm^2^)	64.71±3.87 b	85.97±1.84 a	67.13±4.53 b	48.25±3.87 c
RV- upper (cm^3^)	0.75±0.09 ab	0.99±0.10 a	0.74±0.08 ab	0.58±0.06 b
RL-middle (m)	1.86±0.19 ab	2.15±0.31 a	1.63±0.18 ab	1.08±0.12 b
RA- middle (cm^2^)	21.09±2.23 b	32.85±3.14 a	17.87±1.44 b	15.39±1.59 b
RV- middle (cm^3^)	0.25±0.03 ab	0.32±0.05 a	0.20±0.02 ab	0.18±0.02 b
RL- lower (m)	1.58±0.23 a	1.07±0.17 ab	0.67±0.17 b	0.70±0.08 b
RA-lower (cm^2^)	23.38±2.24 a	25.71±3.27 a	11.63±2.50 b	11.60±1.32 b
RV-lower (cm^3^)	0.37±0.04 a	0.35±0.05 a	0.17±0.04 b	0.16±0.02 b

Data indicate means±SE (*n*=8). Bars labeled with different letters indicate significant differences between treatments (*p*<0.05). SH, shoot height; SBD, stem base diameter; LA, leaf area; CLN, compound leaf number; LWC, leaf water content; AB, aerial biomass; PRL, primary root length (cm); ARD, average diameter of all roots per plant; TRL, total root length; TRSA, total root surface area; TRV, total root volume; TRDW, root biomass; RSM, root to shoot dry mass ratio; RL-upper, root length in upper 0–20cm layer; RA-upper, root surface area in upper 0–20cm layer; RV-upper, root volume in upper 0–20cm layer; RL-middle, root length in the layer between 20 and 40 cm; RA-middle, root surface area in the layer between 20 and 40cm; RV-middle, root volume in the layer between 20 and 40cm; RL-lower, root length in the layer below 40cm; RA-lower, root surface area in the layer below 40cm; and RV-lower, root volume in the layer below 40cm.

For global root traits, there was no significant difference in the average root diameter (ARD) and total root volume (TRV), but the PRL was markedly decreased under all three stresses (Figure 1D; Table 2). Drought increased the total length (TRL), surface area (TRSA), and biomass of the roots (TRDW), whereas salinity and low N had the opposite effect. The root-to-shoot dry mass ratio (RSM) had a markedly higher increase under drought, followed by low N, but did not change under salinity (Table 2).

Root traits at different depths showed different responses to stress treatments (Table 2). Root length (RL-upper) and surface area (RA-upper) in the upper layer and root surface area in the middle layer (RA-middle) were promoted by drought; salinity mainly reduced root growth in the lower section; root traits in both the upper and lower layers were inhibited by low N with the exception of root volume in the upper layer (RV-upper; Table 2).

These results suggested that the growth inhibition of *S. japonica* under low N was stronger than that under salt stress. Drought had a minimal negative effect on the aboveground parts but promoted root growth instead, especially in the upper root system.

### Net Photosynthetic Rate, Chlorophyll Content, and Total N Concentration

**Figure 2:**
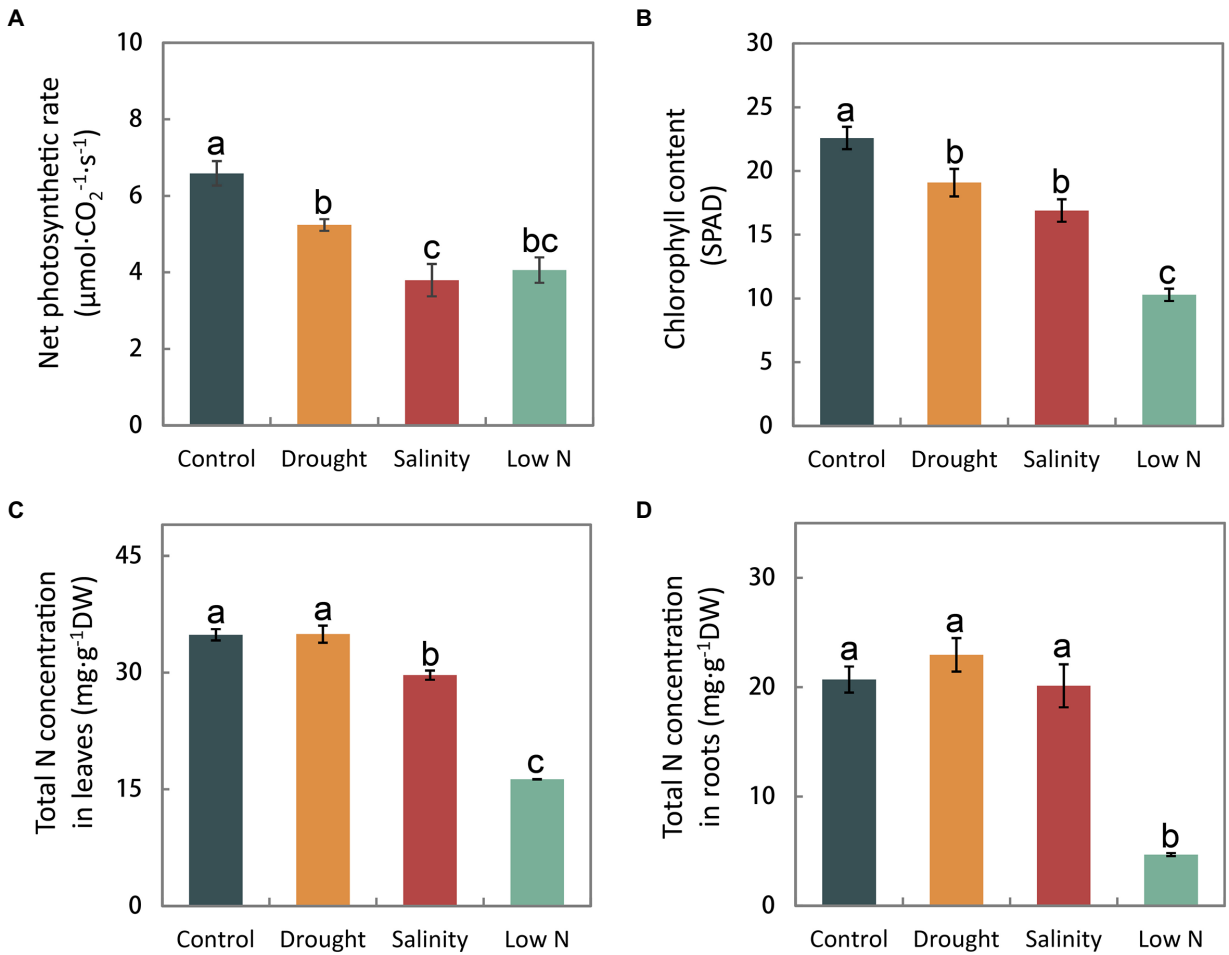
Net photosynthetic rate **(A)**, chlorophyll content **(B)**, and total N concentrations in leaves **(C)** and roots **(D)** of *S. japonica* plants in response to drought, salinity, and low N treatment. Data indicate means±SE of three replicates (12 plants). Bars labeled with different letters indicate significant differences between treatments (*p*<0.05).

In addition to the plant morphology, photosynthesis and nutrient accumulation also distinctly responded to the three abiotic stresses. Both the net photosynthetic rate and the chlorophyll content in the leaves declined under all three treatments (Figures 2A,B). Neither the total N concentrations in leaves nor roots were affected by drought. Nitrogen accumulation decreased only in leaves under salinity, and was greatly reduced in both leaves and roots under low N treatment (Figures 2C,D).

### Accumulation of NH_4_^+^, NO_3_^−^, and NO_2_^−^

**Figure 3:**
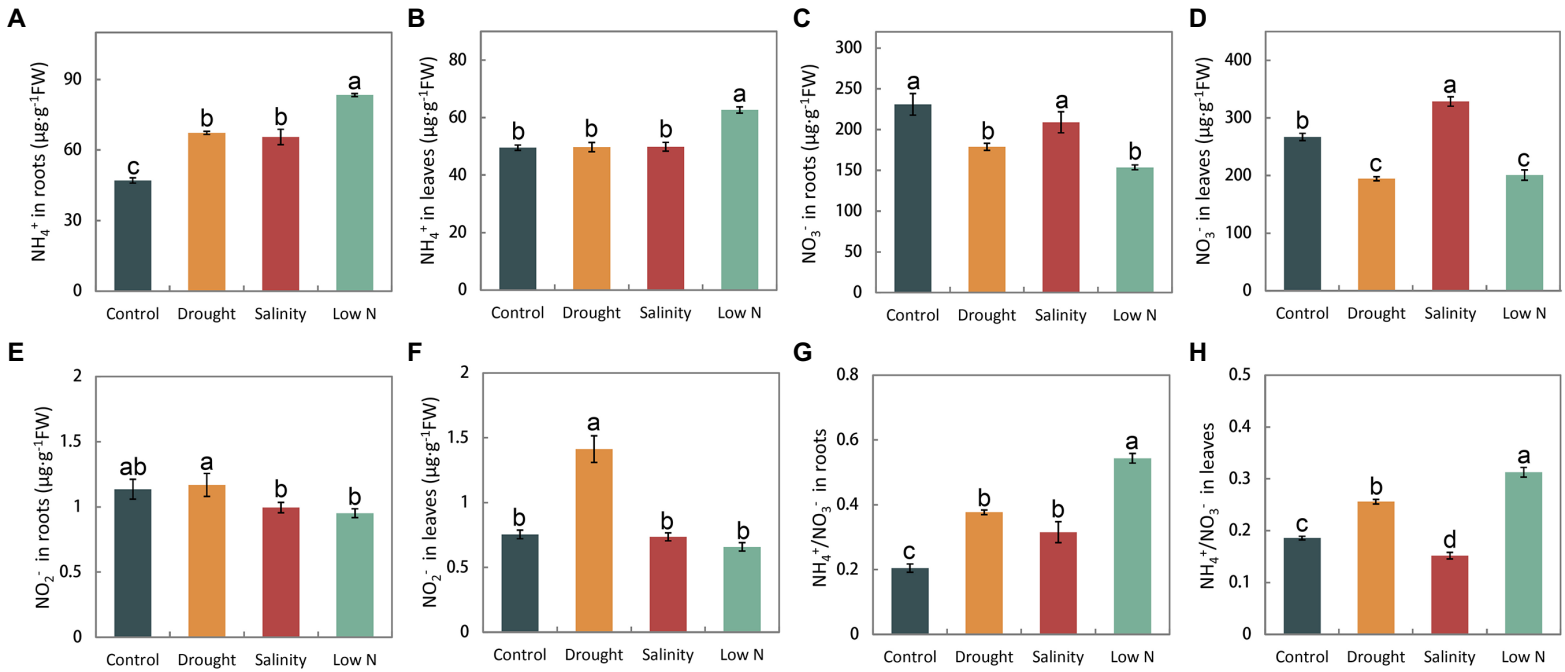
NH_4_^+^, NO_3_^−^, and NO_2_^−^ contents, as well as NH_4_^+^/NO_3_^−^ in roots **(A,C,E,G)** and leaves **(B,D,F,H)** of *S. japonica* as affected by drought, salinity, and low N treatment. Data indicate means±SE of three replicates (12 plants). Bars labeled with different letters indicate significant differences between treatments (*p*<0.05).

We measured the NH_4_^+^, NO_3_^−^, and NO_2_ *^−^* concentrations in the roots and leaves of plants. Stresses caused the NH_4_^+^ concentration to increase in the roots (Figure 3A). Only low N treatment resulted in NH_4_^+^ increases in leaves (Figure 3B). The level of NO_3_^−^ in leaves and roots was reduced in response to drought and low N, but under salt stress, it was not altered in roots and increased in leaves (Figures 3C,D). Moreover, the NO_2_^−^ content was not significantly affected by stresses, with the exception of the increase in leaves under drought (Figures 3E,F). Furthermore, changes in the accumulation of NH_4_^+^ and NO_3_^−^ led to a notable increase in NH_4_^+^/NO_3_^−^ in both leaves and roots under drought and low N treatments, but the increase was higher under low N. However, different from its increase in roots under salinity, the ratio of NH_4_^+^/NO_3_^−^ decreased in leaves when compared with the control level (Figures 3G,H).

### Activity of Enzymes Involved in N Metabolism

**Figure 4:**
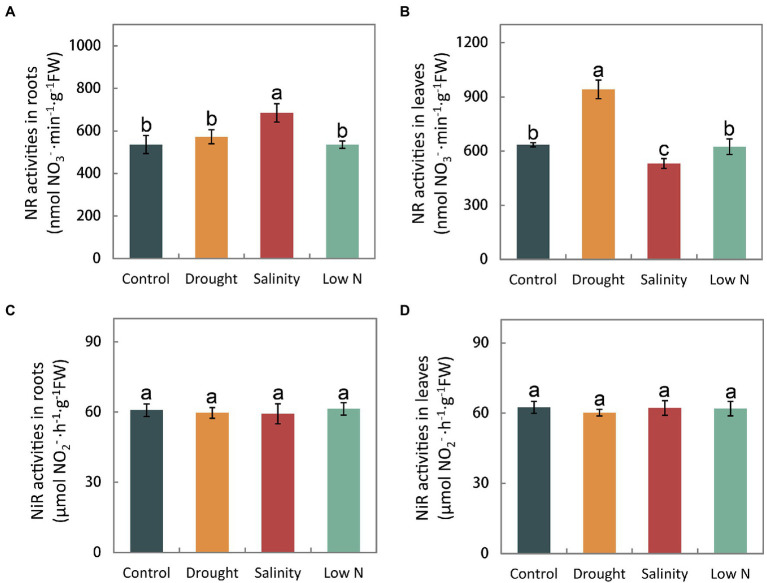
Activities of nitrate reductase and nitrite reductase in roots **(A,C)** and leaves **(B,D)** of *S. japonica* as affected by drought, salinity, and low N treatment. Data indicate means±SE of three replicates (12 plants). Bars labeled with different letters indicate significant differences between treatments (*p*<0.05).

The activity of enzymes, such as NR, NiR, GS, GOGAT, and GDH reflects the process of N assimilation, which occurs after NH_4_^+^ and NO_3_^−^ are taken into roots. In this study, we found that NR activity in the roots under drought and low N was on par with that in the control but increased significantly under salt stress (Figure 4A). NR activity in the leaves was enhanced by drought and inhibited by salinity but was not altered under the low N treatment (Figure 4B). Moreover, all three stress treatments had no effect on NiR activity in either roots or leaves (Figures 4C,D).

**Figure 5:**
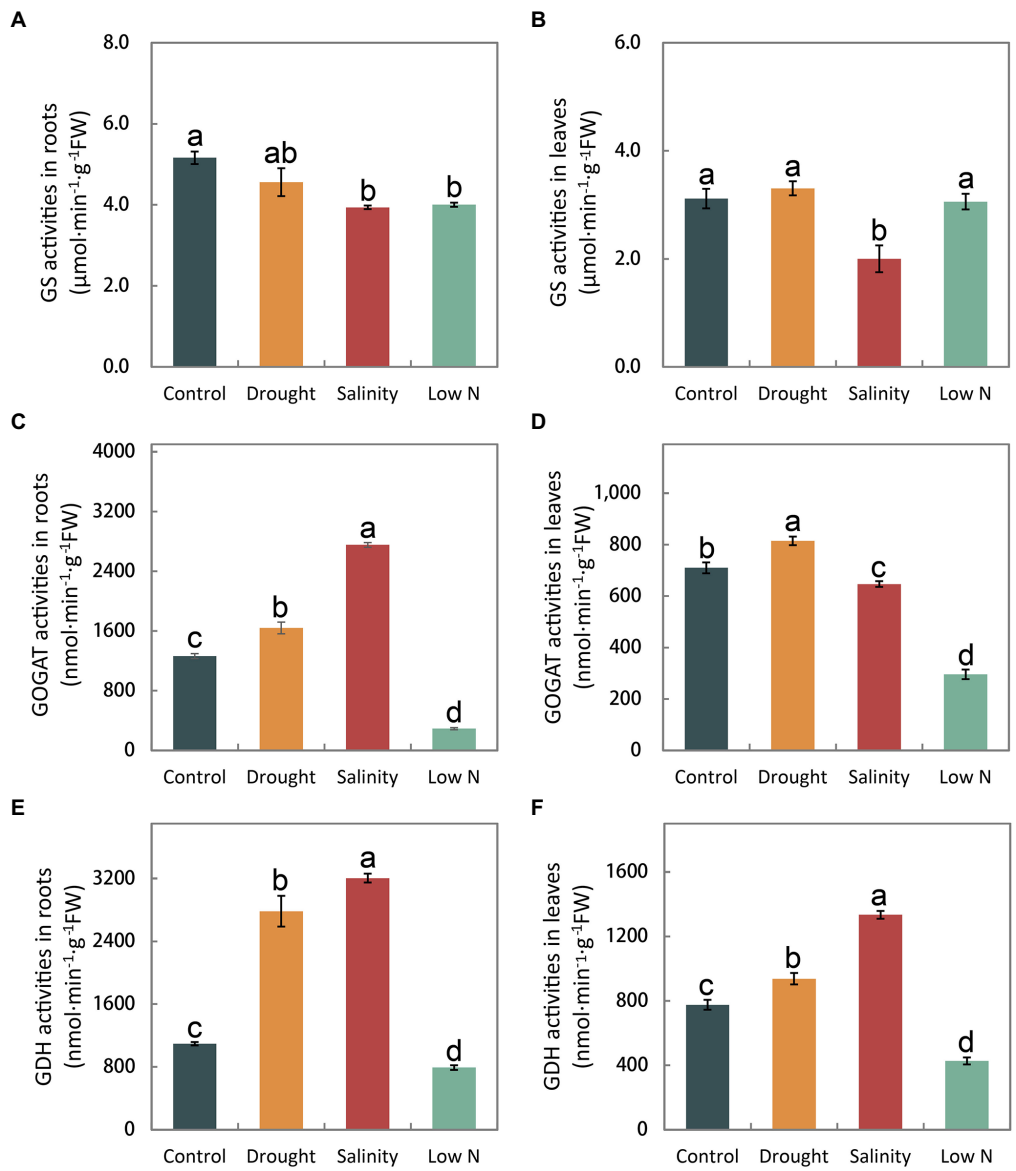
Activities of glutamine synthetase, glutamate synthase, and glutamate dehydrogenase in roots **(A,C,E)** and leaves **(B,D,F)** of *S. japonica* as affected by drought, salinity, and low N treatment. Data indicate means±SE of three replicates (12 plants). Bars labeled with different letters indicate significant differences between treatments (*p*<0.05).

There was no significant difference in the GS activities of roots and leaves under drought, but a reduction was observed under salinity. Low N also decreased GS activity in roots (Figures 5A,B). GOGAT activities increased significantly in both leaves and roots under drought treatment, greatly decreased in both leaves and roots under low N treatment, and highly increased in roots but reduced in leaves under salinity (Figures 5C,D). Additionally, GDH activity in leaves and roots increased significantly under both drought and salinity treatments but more so under salt stress. However, GDH activity was markedly reduced in both roots and leaves in response to low N treatment (Figures 5E,F).

### Transcription Analysis of N-Related Genes

**Figure 6:**
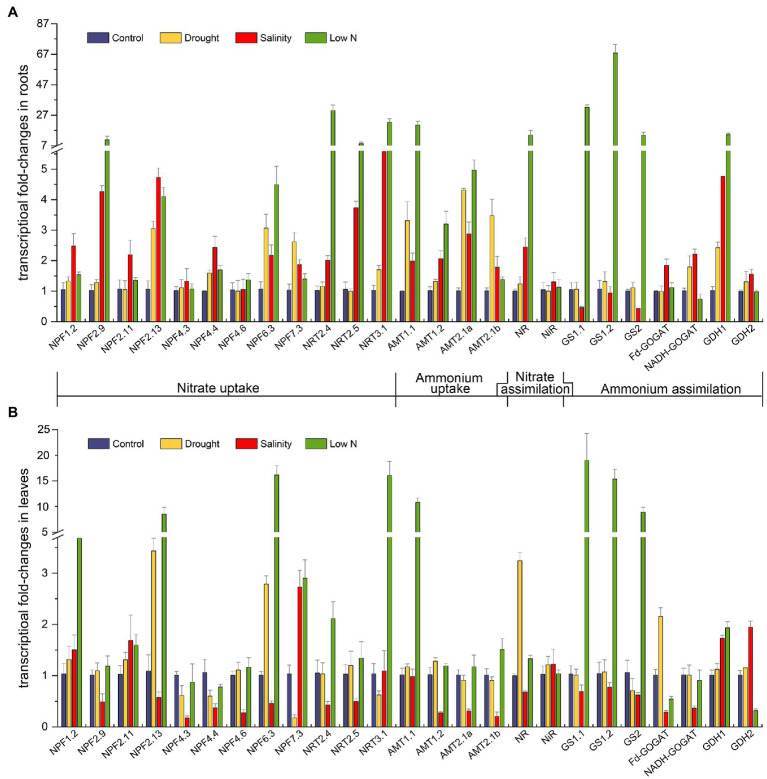
Transcriptional fold-changes of genes involved in N uptake and assimilation in roots **(A)** and leaves **(B)** of *S. japonica* as affected by drought, salinity, and low N treatment. Data indicate means±SE of three replicates (12 plants). Bars labeled with different letters indicate significant differences between treatments (*p*<0.05). *NPF*, nitrate transporter 1(*NRT1*); *NRT2*, nitrate transporter 2; *NRT3*, nitrate transporter 3; *AMT1*, ammonium transporter 1; *AMT2*, ammonium transporter 2; *NR*, nitrate reductase; *NiR*, nitrite reductase; *GS*, glutamine synthetase; *Fd-GOGAT*, ferredoxin-dependent glutamate synthase; *NADH-GOGAT*, NADH-dependent glutamate synthase; and *GDH*, glutamate dehydrogenase.

Key genes implicated in N uptake and metabolism were obtained from the transcriptome data in our previous study (Tian et al., 2021). We performed transcriptional expression analysis of these genes to explore the internal molecular changes of N assimilation in response to different abiotic stresses (Figure 6).

The transcript abundance of *NRTs*, which play key roles in NO_3_^−^ uptake, was diverse. Under drought, the transcript levels of *NRTs* remained unaltered except that *NPF2.13* and *NPF6.3* showed higher expression, and the transcript level of *NPF7.3* increased in the roots but decreased in the leaves (Figures 6A,B). Under salinity treatment, 10 of the 12 *NRTs* in the roots were upregulated, especially *NRT3.1*, but eight of them were downregulated in the leaves (Figures 6A,B). In contrast, the expression of most *NRTs* increased under the low N condition, especially *NPF2.9*, *NPF2.13*, *NPF6.3*, *NRT2.4*, *NRT2.5*, and *NRT3.1* in the roots (Figure 6A) and *NPF1.2*, *NPF2.13*, *NPF6.3*, *NPF7.3*, and *NRT3.1* in the leaves (Figure 6B).

The uptake of NH_4_^+^ is largely determined by specific transporters known as *AMTs*. In the roots, four *AMTs* in our study exhibited higher transcript levels under all three kinds of stresses (Figure 6A). The transcription of *AMT1.1* and *AMT2.1a* ranked as follows: low N>drought>salinity. *AMT1.2* had the highest expression under the low N treatment, and *AMT2.1b* showed the highest expression under drought (Figure 6A). In the leaves, four *AMTs* were not altered by drought; salinity suppressed the mRNA levels of *AMT1.2*, *AMT2.1a*, and *AMT2.1b*, whereas low N strongly induced the expression of *AMT1.1* (Figure 6B).

The imposition of stresses also affected the transcript levels of genes encoding enzymes involved in N metabolism. Drought upregulated the expression of *NADH-GOGAT* and *GDH1* in the roots as well as *NR* and *Fd-GOGAT* in the leaves (Figures 6A,B). Salinity inhibited the transcription of *GS1.1*, *GS1.2*, and *GS2* but enhanced the transcription of *GDH1* and *GDH2* in both roots and leaves. The mRNA levels of *NR*, *Fd-GOGAT*, and *NADH-GOGAT* under salt stress were higher in roots but decreased in leaves (Figures 6A,B). When treated with low N, three genes encoding *GS* and *GDH1* were all intensively induced in roots and leaves, and *NR* also showed extremely high expression in the roots. Finally, *NiR* expression was not affected by any of the three stresses in both roots and leaves (Figure 6).

## Discussion

In this study, we observed that the shoot height, LWC, net photosynthetic rate, and chlorophyll content of *S. japonica* were inhibited under all three treatments (Table 2; Figures 2A,B). Carbohydrates generated through photosynthesis are the main components of plant biomass production. Slowing down plant growth as a result of slower physiological activities such as reduced photosynthetic and transpiration rates may help *S. japonica* seedlings redistribute limited resources, which is an adaptation response in order to enhance survival under stress (Skirycz et al., 2011).

The effects of the three stresses on the root growth of *S. japonica* seedlings were different. As expected, there was better growth of both aboveground parts and roots under drought than under salinity (Table 2). Even when compared with the control, the root systems under drought were more thriving with greater total root length, total root surface area, and root biomass, which were mainly contributed by upper roots (Table 2). Roots are the main organs to absorb water, which can first perceive environmental changes and send chemical signals to the aboveground parts. The extension of the root system depends not only on the species but also on the degree of drought (Jin et al., 2009; Ustun et al., 2018; Alam et al., 2020). The findings showed that *S. japonica* had high adaptation ability to mild drought, which greatly stimulated lateral fine root growth to acquire more water and nutrients. In contrast, sapling growth was most restricted under a low N concentration as indicated by decreases in both shoot and root growth as well as extremely low total N accumulation of the whole plants (Table 2; Figures 2C,D). These data suggest that a low nitrogen concentration of 0.01mM NH_4_NO_3_ cannot meet the requirements of the normal growth of *S. japonica*.

*Sophora japonica* seedlings have distinct internal NH_4_^+^, NO_3_^−^, and NO_2_^−^concentrations in response to drought, salinity, and low N treatments. In our study, all three stresses increased the ratios of NH_4_^+^ and NO_3_^−^ with the exception of leaves under salinity (Figures 3G,H), which may have been because NH_4_^+^ assimilation requires less energy than NO_3_^−^ (Guerrero et al., 1981). Higher NH_4_^+^ concentrations could promote root growth and enhance stress resistance in plants (Fernández-Crespo et al., 2014; Zhang et al., 2014; Ding et al., 2015; Meng et al., 2016; Miranda et al., 2016; Hessini et al., 2020). The reasons for the increase in the ratio of NH_4_^+^ and NO_3_^−^ in plants may be increases in NO_3_^−^ assimilation or NH_4_^+^ uptake and reductions in NH_4_^+^ assimilation or NO_3_^−^ uptake. Based on the changes in NH_4_^+^ and NO_3_^−^ concentrations as well as NR and GS activities in this study (Figures 3A–D, 4A,B, 5A,B), we conclude that the higher ratio of NH_4_^+^ and NO_3_^−^ in roots of *S. japonica* may be caused mainly by (1) reductions in NO_3_^−^ uptake+increases in NH_4_^+^ uptake under drought; (2) increases in NO_3_^−^ assimilation+reductions in NH_4_^+^ assimilation under salinity; and (3) reductions in NO_3_^−^ uptake+reductions in NH_4_^+^ assimilation under low N stress. Additionally, we noted that drought and low N treatment reduced the NO_3_^−^ concentration in both roots and leaves, but unexpectedly, salinity increased the NO_3_^−^ content in leaves and did not affect it in roots (Figures 3C,D). Generally, drought could impair the acropetal translocation of some nutrients, and salinity could suppress NO_3_^−^ absorption because of the competition between Cl- and NO_3_^−^ (Abdelgadir et al., 2005). The results of NO_3_^−^ accumulation under salinity of *S. japonica* may be explained by (i) increases in the NO_3_^−^ concentration compensated osmotically for reductions in soluble carbohydrates when photosynthesis was reduced (McIntyre, 1997); (ii) less absorption of Cl^−^ and promotion of NO_3_^−^ uptake by Na^+^. Similar results were also observed in some halophyte plants (Yuan et al., 2010; Kaburagi et al., 2015). Additionally, (iii) NR activity was inhibited. Obviously, this is not the reason for the unchanged NO_3_^−^ content in roots with a higher increased NR activity. Therefore, we speculated that *S. japonica* may maintain the NO_3_^−^ uptake by reducing Cl^−^ absorption in the roots and store more NO_3_^−^ in vacuoles as an osmotic regulator to promote water absorption by inhibiting NO_3_^−^ assimilation in the leaves to resist salt stress. However, as the levels of Cl^−^ were not measured in the current study, further experiments should be carried out to verify the speculation. In nitrate assimilation process, NO_2_^−^ is produced by converting NO_3_^−^ *via* NR. Our results showed that the changes of nitrite content were inconsistent with that of NR activities (Figures 3E,F, 4A,B). It may be related to non-enzymatic, chemical reduction of nitrite in plants.

Different enzymes involved in N metabolism are dependent on substrates and the flux of inorganic N into organic compounds, which is crucial for N assimilation. The N assimilating enzyme response differs depending not only on the stress type, but also on the plant species, cultivar and analyzed tissue (Meng et al., 2015, 2016; Wang et al., 2019). Nitrate reductase (NR) is the key enzyme of NO_3_^−^ assimilation. Our results showed that NR activities were enhanced in leaves or not affected in roots under drought (Figures 4A,B), which is opposite to the results obtained for *P. simonii* and *M. prunifolia* (Meng et al., 2015; Huang et al., 2018). In contrast, the NR activity remained unchanged under low N in *S. japonica* (Figures 4A,B), similar to previous studies (Meng et al., 2015; Huang et al., 2018). It is thought that higher plants have developed a complex regulatory system controlling NR against environmental factors. Various factors, including NO_3_^−^ itself, carbon and N metabolites, light, phytohormones, CO_2_ concentration, synthesis of the NR protein, and the energy required for the assimilation process including NADH and adenosine triphosphate (ATP), are involved in the regulation of NR activity (Bungard et al., 2002). In *S. japonica*, in addition to the primary signal NO_3_^−^, the induction of NR activity may be affected in other ways, but the underlying mechanism should be further studied.

Glutamine synthetase (GS) and GOGAT are key enzymes of NH_4_^+^ assimilation. GS activities remained unchanged and GOGAT activities increased in both roots and leaves under drought (Figures 5A–D), indicating that *S. japonica* seedlings accelerated the NH_4_^+^ assimilation process to resist drought stress. However, shoot growth was inhibited, which may have been caused by the toxicity of excessive NO_2_^−^ to cells (Figures 1C, 3F). The decreased GS activities in both roots and leaves under salt stress as well as reduced GOGAT activities in both roots and leaves under low N treatment may suggest that the NH_4_^+^ assimilation process was slowed down, eventually resulting in the growth inhibition of both roots and shoots (Figures 5A–D). GDH is considered an alternative enzyme for the GS/GOGAT cycle under abiotic stresses (El-Shora and Abo-Kaseem, 2001). GDH activity increased significantly in the drought and salinity treatments but markedly diminished in response to the low N treatment (Figures 5E,F). Both the aminating and deaminating properties of GDH were considered to be helpful under stress conditions. Amination plays key roles in NH_4_^+^ detoxification and in the replenishment of the glutamate pool, which is largely required to produce protective metabolites such as proline and phytochelatins (Cammerts and Jacobs, 1985; Ashraf et al., 2018). Deamination provides an intermediate to the tricarboxylic acid (TCA) cycle and thus sustained carbohydrate metabolism (Kumar et al., 2000). Abiotic stress severely affected the process of ammonia assimilation in *S. japonica*.

Exposure to stresses also led to changes in gene expression at the molecular level. Prior to NO_3_^−^ assimilation, nitrate is taken up mainly with the help of two types of *NRTs*, low affinity nitrate transporters (NRT1/PTR) and high affinity nitrate transporters (NRT2). In *Arabidopsis*, nitrate transport activity has been proven for 16 out of 53 NRT1/PTR proteins (Léran et al., 2014). Thus, we identified 12 putative nitrate transporters in *S. japonica* through phylogenetic tree and BLAST methods (Supplementary Figure S1), as well as four *AMTs*, one *NR*, one *NiR*, three *GSs*, two *GOGATs*, and two *GDHs* for qRT-PCR. The regulation of these genes might reflect their importance in plant adaptation to various stresses. In our study, the expression of *NPF6.3* was found to be upregulated in roots of *S. japonica* under all the stress treatments (Figure 6A). In *Arabidopsis*, *AtNPF6.3* (*NRT1.1*) not only displays dual affinity for nitrate but also participates in auxin transport and nitrate sensing (Krapp et al., 2014). The upregulation of *SjNPF6.3* may help plants withstand stress by altering root morphology. *AtNPF2.13* (*NRT1.7*) is responsible for transporting nitrate out of older leaves and into younger leaves (Fan et al., 2009). The increases in *SjNPF2.13* transcripts in leaves under drought and low N treatment (Figure 6B) may imply the active process of nitrate source-to-sink remobilization. *NRT1.5* is related to the long-distance transport of nitrate from roots to shoots (Lin et al., 2008). Downregulation of *AtNPF7.3* (*NRT1.5*) has been found to be involved in stress tolerance mechanisms in *Arabidopsis* (Li et al., 2010; Chen et al., 2012). In leaves, the transcript level of *SjNPF7.3* increased under salinity and low N conditions but decreased under drought (Figure 6B). Reduced upward transport of nitrate might be beneficial for root development under drought. It is well known that members of the *NRT2* family play a role in nitrate uptake when the external nitrate concentration is less than 250μM (Crawford and Glass, 1998). It was reported that overexpressing *MdNRT2.4* from apple enhanced low N and osmotic stress tolerance in *Arabidopsis* (Wang et al., 2019); the *nrt2.5* mutation reduced nitrate levels in N-starved *Arabidopsis* (Lezhneva et al., 2014); *AtNRT3;1a* is a key player in a two-component system, which includes *NRT2s*, for nitrate transport (Yong et al., 2010; Kotur et al., 2012). *SjNRT2.4*, *SjNRT2.5*, and *SjNRT3.1* were highly induced in roots by low N treatment in the present study (Figure 6A), indicating their great importance in nitrate uptake and stress resistance in *S. japonica*. Upregulation of most *NRTs* under salt stress (Figure 6A) demonstrated that *S. japonica* had high salinity resistance that could maintain high NO_3_^−^ uptake ability in the roots. Additionally, all three stresses positively regulated the expression of *AMT* genes in the roots of *S. japonica* (Figure 6A). This further proved that *S. japonica* enhanced NH_4_^+^ uptake with less energy consumption than NO_3_^−^ to resist stressful environments, which is consistent with the changes in NH_4_^+^ and NO_3_^−^ contents (Figures 3A,C). In *Puccinellia tenuiflora*, *PutAMT1.1* could mediate toxic MeA, alleviate environmental pH stress, and improve salt tolerance during the early root growth stage after seed germination (Bu et al., 2019). *SjAMT1.1* may perform the same function in leaves under low N treatment. In both the leaves and roots of *S. japonica*, *GS* transcription was more than 10-fold higher than that in the control under low N (Figure 6). As Díaz et al. (2010) stated, proline metabolism is dependent on GS. Overexpression of pine *GS1a* resulted in enhanced tolerance to drought (Molina-Rueda et al., 2013). Strong activation of *GS* would aid *S. japonica* in adapting to a low N environment. The trends of other genes involved in N metabolism were generally consistent with the related enzyme activities.

Collectively, our research is the first to study how the N uptake and metabolism process of *S. japonica* responded to different abiotic stresses at the morphological, physiological, and transcriptional levels. This research greatly advances our understanding of the underlying mechanisms of N uptake and metabolism strategies in response to different abiotic stresses, which could provide insights for stress resistance studies of leguminous tree species. The different responses under the three abiotic stresses provide physiological and molecular basis to evaluate the nitrogen absoption ability of *S. japonica*, which is also the first step to further explore the longivity mechanism of ancient trees. Additionally, this study is also of great significance for maintaining the healthy growth of *S. japonica*, the breeding of resistant varieties, as well as the formulation of feasible management methods.

## Conclusion

The present study provided unique and valuable observations on how the N uptake and metabolism process of *S. japonica* responded to different abiotic stresses at the morphological, physiological, and transcriptional levels. Under drought, root growth was promoted, which may be a result of increases in NH_4_^+^ uptake and NH_4_^+^ assimilation ability. This can be confirmed by the upregulated *AMTs* (*SjAMT1.1*, *SjAMT2.1a*, and *SjAMT2.1b*) and increased GOGAT activity. However, the root growth under stress is influenced by many other factors apart from N uptake and assimilation. Salinity and low N negatively affected plant growth, probably because NH_4_^+^ assimilation was slowed down due to the reduced GS activity. It was found that most *NRTs* were upregulated and the NO_3_^−^ concentration was unaltered even if NR activity increased in roots under salinity, indicating high salinity resistance and that *S. japonica* could retain high NO_3_^−^ uptake ability. Low N treatment most restricted plant growth, which suggested that 0.01mM NH_4_NO_3_ cannot meet the requirements of *S. japonica* for its normal growth. However, plants could strongly activate the expression of genes in the N pathway, especially *NRT2s* and *AMTs*, as well as three *GS* genes to adapt to N limitation. Moreover, *SjNPF2.13*, *SjNPF6.3*, *SjNPF7.3*, and *GDH1* were highly induced by the three treatments. They may play key roles in plant root resistance under environmental stress. All the findings suggested that the resistance of *S. japonica* seedlings to different stresses in our study could be ranked as follows: drought>salinity>low N treatment. The distinct changes in plant growth, N accumulation, enzyme activity, and gene expression indicate that *S. japonica* could plastically regulate N metabolism strategies with the changes of environmental conditions. It provides insights for stress resistance studies of leguminous tree species and long longivity studies of ancient trees. However, further functional identification of the key genes involved in N utilization in response to stress is essential and necessary for maintaining the healthy growth of *S. japonica*, the breeding of resistant varieties, as well as the formulation of feasible management methods.

## Data Availability Statement

The original contributions presented in the study are included in the article/**Supplementary Material**, further inquiries can be directed to the corresponding author.

## Author Contributions

JT and ZZ conceived the study and its design, drafted the manuscript, and revised the paper. JT and YP performed the experiments and contributed to collect and analyze the data. All authors contributed to the article and approved the submitted version.

## Funding

This research was supported by the National Forestry Industry Research Special Funds for Public Welfare Projects (China; 201404302). The funders had no role in study design, data collection and analysis, decision to publish, or preparation of the manuscript.

## Conflict of Interest

The authors declare that the research was conducted in the absence of any commercial or financial relationships that could be construed as a potential conflict of interest.

## Publisher’s Note

All claims expressed in this article are solely those of the authors and do not necessarily represent those of their affiliated organizations, or those of the publisher, the editors and the reviewers. Any product that may be evaluated in this article, or claim that may be made by its manufacturer, is not guaranteed or endorsed by the publisher.

## Footnotes

^1^ https://www.arabidopsis.org/
