# Supplementary material for: Drought, Salinity, and Low Nitrogen Differentially Affect the Growth and Nitrogen Metabolism of Sophora japonica (L.) in a Semi-Hydroponic Phenotyping Platform
Source: Front Plant Sci. 2021 Oct 1;12:715456. doi: 10.3389/fpls.2021.715456 (PMC8522681; doi:10.3389/fpls.2021.715456)
Supplement: Supplementary file 1 [file Data_Sheet_1.ZIP › Supplementary file/Figure S1.docx]

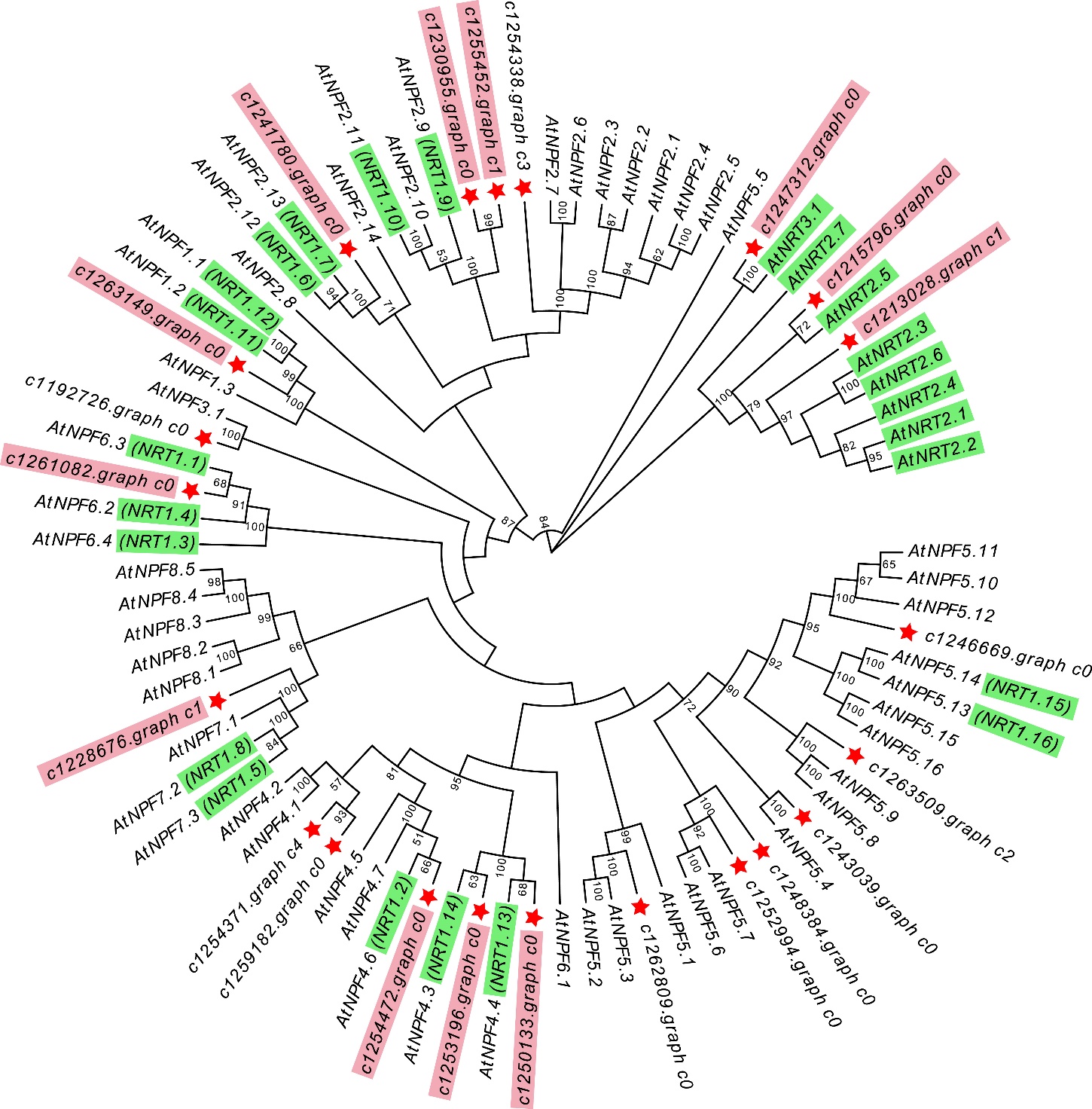


Figure S1. Phylogenetic analysis of NTRs in S. japonica with all NRT genes in Arabidopsis. The protein sequences are provided in Table S1 and Table S2. The green boxes represent the genes that were proven to transport nitrate in Arabidopsis. The pink boxes represent the genes selected for quantitative real-time PCR analysis in S. japonica at present study.
